# Effects of *Septoglomus constrictum* and *Bacillus cereus* on the competitive growth of *Ageratina adenophora*

**DOI:** 10.3389/fmicb.2023.1131797

**Published:** 2023-06-02

**Authors:** Ewei Du, Yaping Chen, Yang Li, Yahong Li, Zhongxiang Sun, Ruoshi Hao, Furong Gui

**Affiliations:** ^1^State Key Laboratory for Conservation and Utilization of Bioresources in Yunnan, College of Plant Protection, Yunnan Agricultural University, Kunming, China; ^2^Graduate School, Yunnan Agricultural University, Kunming, China; ^3^Yunnan Plant Protection and Quarantine Station, Kunming, China; ^4^Yunnan Plateau Characteristic Agriculture Industry Research Institute, Kunming, China

**Keywords:** *Ageratina adenophora*, arbuscular mycorrhizal fungi, *Bacillus*, synergistic inoculation, competitive advantage

## Abstract

Beneficial microorganisms play a pivotal role in the invasion process of exotic plants, including arbuscular mycorrhizal fungi (AMF) and *Bacillus*. However, limited research exists on the synergistic influence of AMF and *Bacillus* on the competition between both invasive and native plants. In this study, pot cultures of *Ageratina adenophora* monoculture, *Rabdosia amethystoides* monoculture, and *A. adenophora* and *R. amethystoides* mixture were used to investigate the effects of dominant AMF (*Septoglomus constrictum*, SC) and *Bacillus cereus* (BC), and the co-inoculation of BC and SC on the competitive growth of *A. adenophora*. The results showed that inoculation with BC, SC, and BC + SC significantly increased the biomass of *A. adenophora* by 14.77, 112.07, and 197.74%, respectively, in the competitive growth between *A. adenophora* and *R. amethystoides*. Additionally, inoculation with BC increased the biomass of *R. amethystoides* by 185.07%, while inoculation with SC or BC + SC decreased *R. amethystoides* biomass by 37.31 and 59.70% compared to the uninoculated treatment. Inoculation with BC significantly increased the nutrient contents in the rhizosphere soil of both plants and promoted their growth. Inoculation with SC or SC + BC notably increased the nitrogen and phosphorus contents of *A. adenophora*, therefore enhancing its competitiveness. Compared with single inoculation, dual inoculation with SC and BC increased AMF colonization rate and *Bacillus* density, indicating that SC and BC can form a synergistic effect to further enhance the growth and competitiveness of *A. adenophora*. This study reveals the distinct role of *S. constrictum* and *B. cereus* during the invasion of *A. adenophora*, and provide new clues to the underlying mechanisms of interaction between invasive plant, AMF and *Bacillus*.

## 1. Introduction

The invasion of exotic plants has resulted in a rapid decline in global biodiversity (Powell et al., 2013), with severe implications on the functioning of the whole ecosystem function (Richardson and Pyšek, 2012) and incurring significant economic losses. Thus, understanding the invasion mechanisms of these plants has emerged as a pressing and pragmatic concern (Sol et al., 2012; Anna et al., 2020). The spreading capacity in their new habitat is at least partly influenced by their association with symbiotic microorganisms (Fahey and Stephen, 2022). Beneficial symbiotic microorganisms regulate competitive growth between invasive and native plants (Abbott et al., 2015; Dawson et al., 2016). The Enhanced Mutualisms Hypothesis (EMP) suggests that invasive plants facilitate positive soil feedback by enriching their associated beneficial symbiotic microbes, thereby promoting their own growth and expansion (D’Antonio et al., 2017; Zhang et al., 2020). In contrast, the Degraded Mutualisms Hypothesis (DMP) proposes that exotic plants can weaken the symbiotic relationship between beneficial microbes and native plants, creating a competitive disadvantage for the latter and facilitating the successful invasion of invasive plants (Vogelsang and Bever, 2009).

Arbuscular mycorrhizal fungi (AMF) can form a mutualistic symbiosis with most terrestrial plants to adapt better to limited nutrient supply conditions (Smith and Smith, 2011). In most nutrient-poor soils, the low availability of nitrogen and phosphorus is often the main limiting factor affecting plant growth (Xu et al., 2012). AMF is reported to dominate the uptake of phosphate, ammonium and nitrate nitrogen uptake in symbiotic plants (Fan et al., 2020; Wang et al., 2020; Xie et al., 2022). Accumulating evidence suggests that AMF can develop symbiosis with invasive plants, which greatly contributes to the successful invasion of invasive plants (Lekberg et al., 2013; Aslani et al., 2019). AMF enhances the resistance of invasive plants to biotic and abiotic stresses, thus promoting the establishment of invasive plants in their new habitats (Dickie et al., 2017; Qi et al., 2022). The exotic plants affect AMF abundance and richness (Zhang et al., 2017; Kong et al., 2022). When invasive plants grow alongside native plants, the mycorrhizal colonization rate of the invasive plants increases while that of the native plants decreases, giving invasive plants a competitive advantage over native plants (Zhang et al., 2018). AMF forms a common mycorrhizal network (CMN) that allows the plant-to-plant transfer of carbon and mineral nutrients between native plants and alien plants (Weremijewicz et al., 2018; Awaydul et al., 2019). Therefore, AMFs are more beneficial to invasive plants than native species, which may be a key factor in the successful invasion of exotic plants.

*Bacillus* is an important plant growth-promoting rhizobacteria (PGPR). Most *Bacillus* have many beneficial effects on plants and can promote plant growth by activating soil nutrients and producing phytohormones (Fan et al., 2018; Saxena et al., 2020), which also protects plants from biotic stresses by directly inhibiting plant pathogens and inducing plants to acquire systemic resistance (Stefany et al., 2021; Kurniawan and Chuang, 2022). *Bacillus* diversity and abundance differed in the rhizosphere soil of invasive and native plants. Chen et al. (2022) found that *Bacillus* diversity differed in the rhizosphere soil of the exotic (*Flaveria bidentis*) and native plants (*Setaria viridis*). The relative abundance of *Brecibacterium frigoritolerans* was higher in the *F. bidentis* rhizosphere than in the *S. viridis* rhizosphere. Additionally, the dominant *Bacillus* in the *F. bidentis* rhizosphere promoted *F. bidentis* competitive growth by elevating soil nitrogen and phosphorus levels. These studies indicate that *Bacillus* is also crucial for the successful invasion of exotic plants. As previously evidenced, AMF and PGPR can mutually promote their own growth and development, forming a synergistic effect to further enhance plant growth and promote their tolerance to diverse stresses (Krishnamoorthy et al., 2016; Hidri et al., 2019). However, few studies have examined whether combinations of AMF and *Bacillus* would contribute to the successful invasion of invasive plants. Therefore, understanding the effects of AMF, *Bacillus* and their combinations in the rhizosphere soil on the competitiveness of invasive plants with native plants is conducive to exploring the ecological role of two functional rhizosphere microorganisms in plant invasion.

*Ageratina adenophora* (Spreng.), a perennial herbaceous plant of the Asteraceae family, originates from Mexico and Costa Rica. Due to its strong reproductive and dispersal capabilities, morphological plasticity and stress tolerance, it has invaded many countries across Asia, Africa, and Oceania (Poudel et al., 2019; Tang et al., 2019; Gu et al., 2021). Since its first introduction from Myanmar into the Yunnan Province of China in the 1940s, *A. adenophora* has spread widely across southwestern and central China, becoming a dominant and invasive plant in China (Wang and Wang, 2006; Gui et al., 2009). *A. adenophora*’s colonization in southwestern China expanded considerably, with its suitable habitat distribution rapidly advancing eastward and northward (Li W. T. et al., 2022). The invasion of this plant has resulted in the destruction of native biodiversity, alteration of ecological community structures, and posed a severe threat to the development of agriculture, forestry and livestock industries, resulting in enormous economic and ecological losses (Song et al., 2017; Wang et al., 2017; Ren et al., 2021). *A. adenophora* has been shown to selectively aggregate functional microbes that mediate soil nutrient cycling to form a favorable soil microenvironment in the invasive habitats that facilitate its invasion (Niu et al., 2007; Zhao et al., 2007; Li Q. et al., 2022). In contrast to native plant rhizosphere soil, *A. adenophora* rhizosphere soil exhibits a remarkably higher abundance of nitrogen-fixing bacteria, phosphorus-solubilizing bacteria, and IAA-producing bacteria (Xu et al., 2012; Fang et al., 2019). Our previous studies demonstrated that *Bacillus cereus* has a high abundance in the rhizosphere soil of *A. adenophora* and *R. amethystoides* and exerted a positive feedback effect on *A. adenophora* (Sun et al., 2021; Du et al., 2022a). The AM fungus *Septoglomus constrictum* was also identified in the rhizosphere soil of *A. adenophora* and *R. amethystoides*, which improved the growth of *A. adenophora* and its resistance to *A. gossypii* feeding (Yu et al., 2012; Li et al., 2016; Du et al., 2022b). However, the relationship between AMF and *Bacillus* in the rhizosphere of *A. adenophora* and *R. amethystoides* and the effects of this relationship on the competitive growth between the two plants remain unknown.

To address this knowledge gap, we hypothesized that a synergistic interaction between *B. cereus* and *S. constrictum* might enhance the competitive growth of *A. adenophora*. To test this hypothesis, we compared the effects of single inoculation with AMF or *Bacillus* and co-inoculation of two kinds of microorganisms on the AMF colonization rate and *Bacillus* density of *A. adenophora* to explore whether both microorganisms form a synergistic effect. To illustrate the impact and reasons of microorganisms on plant growth, we compared their biomass, relative competitiveness, root growth characteristics, and nutrient content, as well as the soil’s available nutrient content.

## 2. Materials and methods

### 2.1. Microbial inoculation preparation

The spores of *S. constrictum* were isolated from the rhizosphere soil of *A. adenophora* in our previous study (Du et al., 2022b). The mycorrhizal inoculum, consisting of fragments of colonized roots, spores, and hyphae of *S. constrictum,* was propagated using maize as the host plant. The spore density (20 spores/100 g of soil) was determined based on the quantity of *S. constrictum* spores in the rhizosphere soil of *A. adenophora* in the field and used for subsequent analyses.

The *B. cereus* strain A20 (GenBank accession: OM149794) was isolated from *A. adenophora* and *R. amethystoides* rhizosphere soil (Du et al., 2022a). The strain’s organic phosphate-solubilizing ability, inorganic phosphate-solubilizing ability, potassium-solubilizing ability, nitrogen-fixing ability, and IAA-producing ability were 53.66, 92.38, 51.33, 23.67, 61.55 mg/L, respectively. The siderophores-producing ability of the strain was 0.48, according to the methodology of Payne (1994). The strain was separately cultured on nutrient agar plates at 37°C for 8–12 h to obtain single colonies. The colonies of activated *Bacillus* were selected using an aseptic toothpick and incubated in 1 mL of nutrient liquid medium culture in a 1.5 mL centrifuge tube. After shaking at 180 rpm for 24 h at 37°C, the liquid was transferred into a triangular flask containing 100 mL of nutrient liquid broth medium and shaken at 180 rpm for 24 h at 37°C. The *Bacillus* suspension was then expanded to a concentration of 10^8^ CFU/mL (Sun et al., 2022).

### 2.2. Plants and soil preparation

The soils and seeds of the exotic and native plants were purchased from Yunnan Agricultural University (Kunming, China; 25°08′30″ N, 102°45′13″ E, altitude 1964 m). Before sowing, the seeds were surface disinfected for 10 min in a 5% sodium hypochlorite solution. Next, the seeds were washed 5 times using sterile water, followed by 1 min soaking in 75% alcohol and rinsing 5 times in sterile water. The soil was crushed, sieved (2 mm), then mixed with vermiculite (v/v = 1:1) [(Mg, Fe, Al)_3_[(Si,Al)_4_O_10_(OH)_2_]. 4H_2_O] (Dounan Plant and Flower Co., Ltd., Kunming, China). The properties of the soil were as follows: pH = 6.25, 15.502 g/kg organic matter, 0.899 g/kg total nitrogen, 0.351 g/kg total phosphorus, 40.03 g/kg total potassium, 20.28 μg/g available nitrogen, 5.089 μg/g available phosphorus (AP), and 32.32 mg/kg available potassium (AK). Lastly, the mixtures were subjected to 2 h heating (121°C) in an autoclave.

### 2.3. Experiment design

The impact of AMF and *Bacillus* on competitive *A. adenophora* and *R. amethystoides* growth was investigated by conducting a greenhouse experiment at the Yunnan Agricultural University. The experiment considered two factors: (1) Plant type: *A. adenophora*, *R. amethystoides*, *A. adenophora* and *R. amethystoides*, and (2) inoculum treatments: C (uninoculated treatment), BC (inoculated with *B. cereus*), SC (inoculated with *S. constrictum*), and BC + SC (dual-inoculation with *B. cereus* and *S. constrictum*). Following the design of Gibson et al. (1999) and Zhang et al. (2018), the planting included a monoculture of *A. adenophora* and *R. amethystoides*, and *A. adenophora* and *R. amethystoides* mixture, with two plants per pot for monoculture treatment, and one *A. adenophora* and one *R. amethystoides* per pot for mixture treatment. Before starting the experiment, 1 kg soil in the pots was used for sowing the seeds of *A. adenophora* and/or *R. amethystoides* and inoculums of *S. constrictum* (20 per/100 g of soil) and/or *B. cereus* (10 mL 10^8^ CFU/mL). For the non-AMF treatment, we added the same amount of sterilized inoculum and the filtrate (<20 mm) of the AMF inoculum, while for the non-*Bacillus* treatment, we added 10 mL of the sterilized bacterial suspension. The study was conducted using a randomized design comprising 10 repetitions per treatment (3 planting treatments × 4 inoculation treatments × 10 replicates = 120 pots). The plants were watered with sterile water once every 2 days, and the seeds were grown in a controlled environment in a greenhouse at a temperature of 25°C with 10 h light/14 h dark.

### 2.4. Measurement

#### 2.4.1. Biomass and corrected index of relative competition intensity

*Ageratina adenophora* and *Rabdosia amethystoides* were collected under different treatments following germination for 120 days. The soil in the roots and shoots was collected to measure their biomass. All roots and shoots were dried (80°C, 72 h), and the growth index data were obtained. The total biomass (aboveground and belowground biomass) was measured. The plant competitiveness was quantified using CRCI, calculated following the methodology of Oksanen et al. (2006):

CRCI = arcsin [(X – Y) / max (X, Y)],

where X and Y represent individual plant biomass in intraspecific and interspecific competition, respectively; CRCI value >0 represents the negative effect, whereas CRCI value <0 represents the positive effect of the competition on the target plant.

#### 2.4.2. Root growth characteristics

The roots were washed, cut, and evenly distributed in a scanning tray filled with water. They were scanned using a root scanner (Epson Expression 10000XL; Epson, Long Beach, CA, United States) and analyzed using the WinRhizo software (Regent Instruments Inc., Québec City, QC, Canada) and their root length (RL), root surface area (RS), root diameter (RD), and root volume (RV) were calculated.

#### 2.4.3. Total nitrogen, phosphorus and potassium concentration

The dried plants were ground with a high-throughput Tissuelyser-48 grinder (Shanghai Jingxin Industrial Development Co., Ltd. Shanghai, China). C concentration was determined using 20 mg plant powder via the potassium dichromate-concentrated sulfuric acid oxidation method (K2Cr2O7-H2SO4) (Kong et al., 2022). The plant samples (2 g powder) were digested in a concentrated perchloric and nitric acids mixture (v:v = 1: 6) to measure the N, P, and K concentrations. Nitrogen and phosphorus content was analyzed separately using the micro-Kjeldahl method (Nelson and Sommers, 1973) and inductively coupled plasma spectroscopy (Isaac and Johnson, 1983). Six replicates were set up for each treatment.

#### 2.4.4. Colonization of AMF

After rinsing in 10% KOH, the roots were acidified with 2% HCl and stained with 0.1% acid fuchsin solution. Then, the mycorrhizal root colonization percentage was determined by visually observing fungal colonization (Zhang et al., 2017). The magnified intersections method was used for analyzing AMF colonization in the *A. adenophora* and *R. amethystoides* roots (Giovannetti and Mosse, 1980; Biermann and Linderman, 1981). Two hundred root segments for each replicate were analyzed using the Olympus BX43 compound microscope (Olympus, Tokyo, Japan), and six replicates per treatment were conducted. The colonization percentage of each segment was measured by colonization (presence of hyphae, vesicles, or arbuscules) in that region. AMF colonization was calculated by combining the percentage colonization of the 200 root segments.

#### 2.4.5. Density of *Bacillus cereus*

The density of *B. cereus* in each soil sample was analyzed to investigate different treatment impacts on their growth on nutrient agar medium plates using the suspension dilution method (Yang et al., 2012; Du et al., 2020; Sun et al., 2022). Briefly, 1 g rhizosphere soil with 9 mL of sterile water was incubated at 200 r/min, heated at 90°C for 10 min, and serially diluted. Then, 0.1 mL of 10^−3^ supernatant was added to nutrient agar plates and cultured for 18 h at 37°C. Colonies were counted as the colony-forming units/ per gram of dry soil (CFU/g) according to volume dilution. Six replicates were conducted for each treatment.

### 2.5. Statistical analysis

The variables are expressed as mean ± standard error (*n* = 6). The SPSS v21.0 (IBM, Armonk, New York) software was used for statistical analyses. The Shapiro–Wilk test was used for testing data normality. All data conformed to normality distribution. A two-way ANOVA (Tuckey test) was conducted to determine inoculum effects on the biomass, root growth characteristics, total N, P, and K concentrations, and soil characteristics in the monoculture and mixture treatment. Differences among different inoculums (C, BC, SC, and BC + SC) in these variables above were determined using multiple comparisons (Tuckey test). A one-way ANOVA (Tuckey test) was conducted to determine inoculum effects on CRCI, AMF colonization rate, and *Bacillus* density in the monoculture and mixture treatment. Monoculture and the mixture influence on plant growth were evaluated using Student’s *t*-test. Pearson’s rank correlation coefficient was used to analyze the correlation between AMF colonization and plant growth parameters. All graphics were created by Excel and Origin 2019 (OriginLab, United States).

## 3. Results

### 3.1. Impacts of competition and inoculum on *Ageratina adenophora* and *Rabdosia amethystoides* biomass

**Figure 1:**
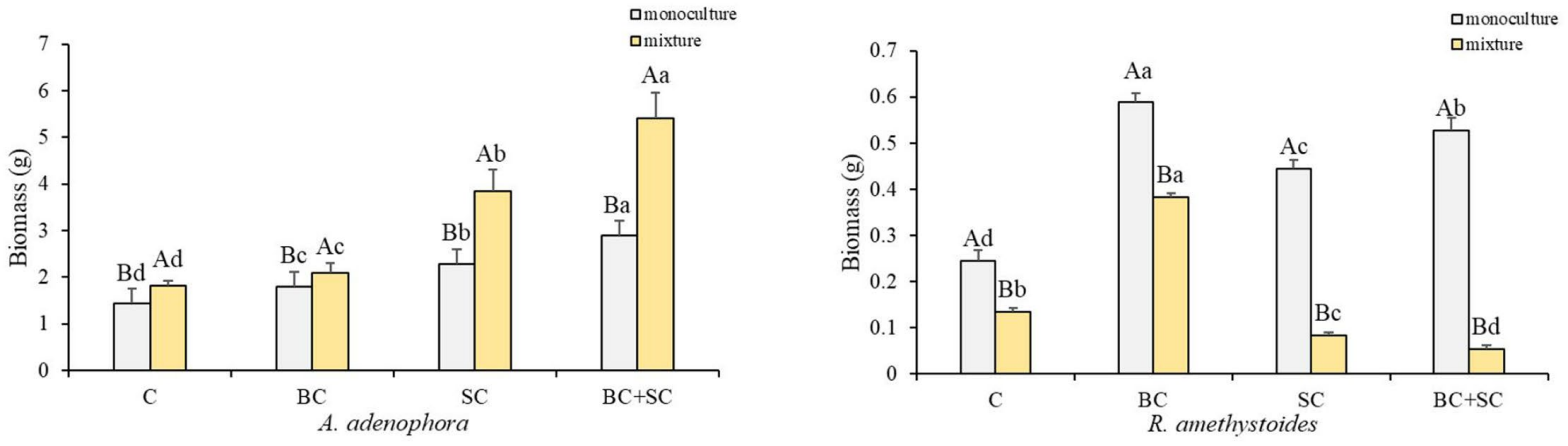
Effect of competition and inoculum on *A. adenophora* and *R. amethystoides* biomass. C, control; BC, inoculated with *B. cereus*; SC, inoculated with *S. constrictum*; BC + SC, inoculated with *B. cereus* and *S. constrictum*. Different lowercase letters in lower case indicate significant differences between the four treatments at *p* < 0.05. Different uppercase letters indicate significant differences between the monoculture or mixture at *p* < 0.05. Error bars represent ± SE of mean (*n* = 6).

The present study investigates the impact of competition on the biomass of two plant species, *A. adenophora* and *R. amethystoides*, and the effect of inoculation treatments on their growth (Figure 1 and Supplementary Table S1). Our results demonstrate that competition has an opposite effect on the two species. *A. adenophora* exhibited a significantly higher biomass in the presence of other plants [C: *F*_(1,10)_ = 0.288; BC: *F*_(1,10)_ = 2.164; SC: *F*_(1,10)_ = 3.501; BC + SC: *F*_(1,10)_ = 21.928; all *p* < 0.001], whereas *R. amethystoides* showed a higher biomass in monoculture [C: *F*_(1,10)_ = 1.173; BC: *F*_(1,10)_ = 5.483; SC: *F*_(1,10)_ = 2.564; BC + SC: *F*_(1,10)_ = 1.342; all *p* < 0.001]. The inoculation treatments significantly increased the biomass of both species, regardless of the presence [*F*_(3,20)_ = 124.773, *p* < 0.001] or absence of competition [*F*_(3,20)_ = 113.963, *p* < 0.001]. In particular, the BC + SC treatment showed the greatest positive impact, increasing *A. adenophora* biomass by 197.74 and 116.39% in the mixture and monoculture treatments, respectively. Interestingly, the effect of inoculation treatments varied depending on the presence of competition. The biomass of *R. amethystoides* in the mixture treatment was significantly reduced by the SC and BC + SC treatments, while the BC treatment led to a remarkable increase. In contrast, all inoculation treatments positively impacted the biomass of *R. amethystoides* in the monoculture treatment. These results suggest that competition may modulate the response of plants to inoculation treatments.

### 3.2. Impacts of inoculum on the corrected index of relative competition intensity of *Ageratina adenophora* and *Rabdosia amethystoides*

**Figure 2:**
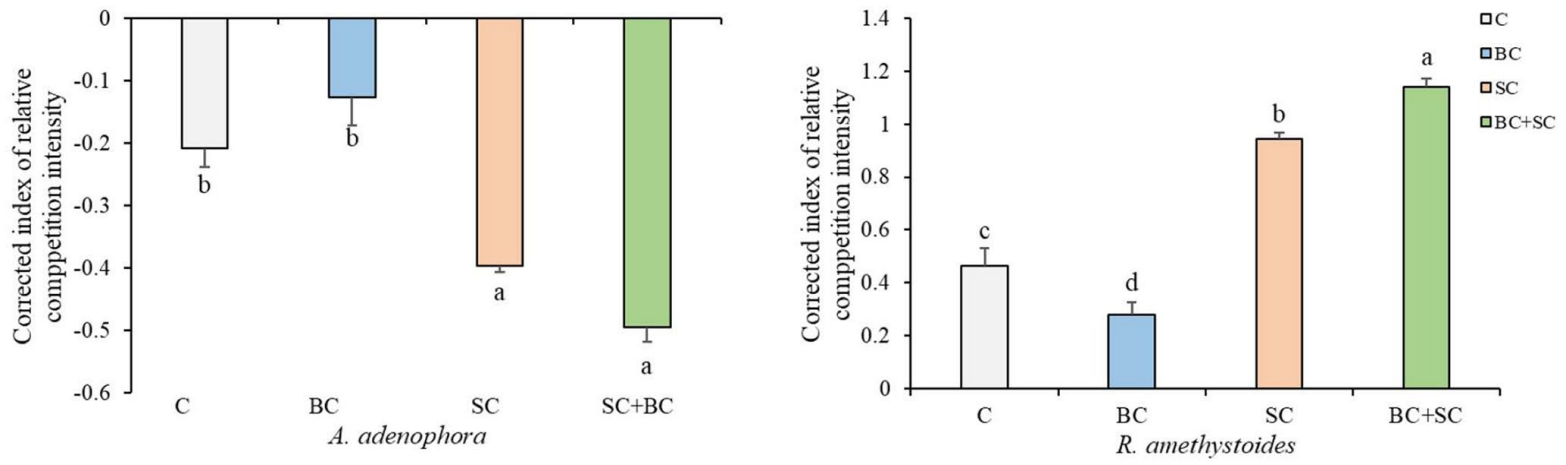
Effect of inoculum on *A. adenophora* and *R. amethystoides* CRCI. C, control; BC, inoculated with *B. cereus*; SC, inoculated with *S. constrictum*; BC + SC, inoculated with *B. cereus* and *S. constrictum*. Different lowercase letters in lower case indicate significant differences between the four treatments at *p* < 0.05. Error bars represent ± SE of mean (*n* = 6).

Our results demonstrate that interspecific competition has a significant positive effect on *A. adenophora* growth [*F*_(3,20)_ = 31.547, *p* < 0.001] and a significant negative effect on *R. amethystoides* growth [*F*_(3,20)_ = 492.927, *p* < 0.001] in both non-inoculation and inoculation treatments (Figure 2). Additionally, inoculation with BC reduced the positive effect of interspecific competition on *A. adenophora* (*p* = 0.251), while inoculation with SC and BC + SC enhanced the positive effect on *A. adenophora* (SC: *p* = 0.001; SC + BC: *p* < 0.001). Inoculation of BC treatment alleviated the negative effect on *R. amethystoides* growth when grown with *A. adenophora* in comparison with the control (*p* < 0.001), while inoculation with SC and BC + SC treatments enhanced this negative effect (SC: *p* < 0.001; SC + BC: *p* < 0.001).

### 3.3. Impacts of competition and inoculum on root growth characteristics of *Ageratina adenophora* and *Rabdosia amethystoides*

**Figure 3:**
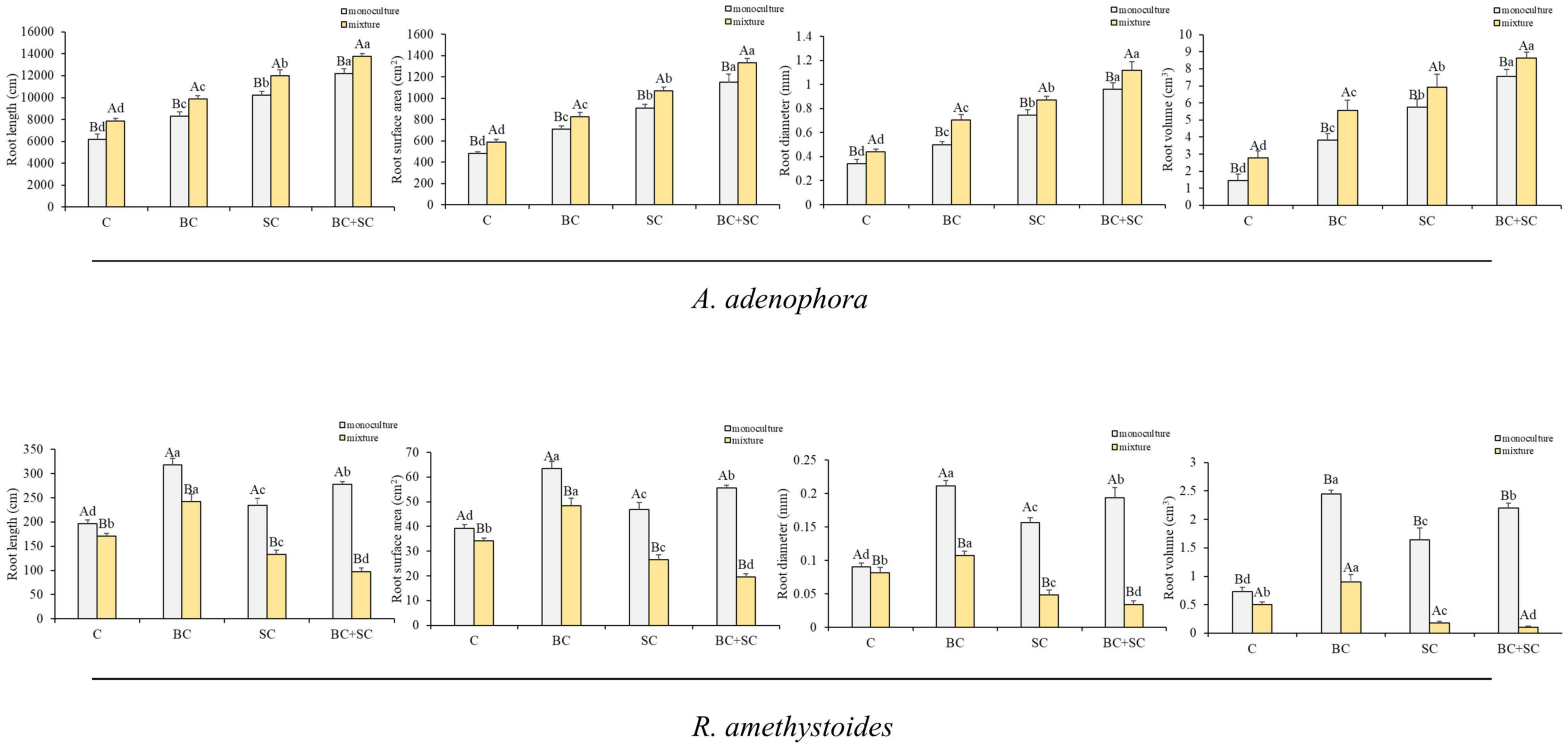
Effect of competition and inoculum on the root growth characteristics of *A. adenophora* and *R. amethystoides*. C, control; BC, inoculated with *B. cereus*; SC, inoculated with *S. constrictum*; BC + SC, inoculated with *B. cereus* and *S. constrictum*. Different lowercase letters significant differences between the four treatments at *p* < 0.05. Different uppercase letters indicate significant differences between the monoculture or mixture at *p* < 0.05. Error bars represent ± SE of mean (*n* = 6).

The effect of competition on root length, root surface area, root diameter and root volume of *A. adenophora* and *R. amethystoides* were consistent with the biomass trend (Figure 3 and Supplementary Table S1). Monoculture treatment of *A. adenophora* led to significantly lower root growth characteristics than mixture treatment [C: root length: *F*_(1,10)_ = 3.478; root surface area: *F*_(1,10)_ = 2.822; root diameter: *F*_(1,10)_ = 3.451; root volume: *F*_(1,10)_ = 0.074; BC: root length: *F*_(1,10)_ = 0.564; root surface area: *F*_(1,10)_ = 0.277; root diameter: *F*_(1,10)_ = 5.739; root volume: *F*_(1,10)_ = 0.542; SC: root length: *F*_(1,10)_ = 0.645; root surface area: *F*_(1,10)_ = 0.065; root diameter: *F*_(1,10)_ = 0.273; all *p* < 0.001; root volume: *F*_(1,10)_ = 3.259, *p* = 0.009; BC + SC: root length: *F*_(1,10)_ = 1.695, *p* < 0.001; root surface area: *F*_(1,10)_ = 6.935, *p* = 0.001; root diameter: *F*_(1,10)_ = 1.291, *p* = 0.002; root volume: *F*_(1,10)_ = 0.089, *p* = 0.001], while monoculture treatment of *R. amethystoides* was associated with significantly higher root growth characteristics compared to mixture treatment [C: root length: *F*_(1,10)_ = 1.127, *p* < 0.001; root surface area: *F*_(1,10)_ = 1.217, *p* < 0.001; root diameter: *F*_(1,10)_ = 1.135, *p* = 0.049; root volume: *F*_(1,10)_ = 2.646; BC: root length: *F*_(1,10)_ = 13.136; root surface area: *F*_(1,10)_ = 11.633; root diameter: *F*_(1,10)_ = 0.178; root volume: *F*_(1,10)_ = 4.546; SC: root length: *F*_(1,10)_ = 1.483; root surface area: *F*_(1,10)_ = 1.348; root diameter: *F*_(1,10)_ = 2.248; root volume: *F*_(1,10)_ = 1.613; BC + SC: root length: *F*_(1,10)_ = 1.839; root surface area: *F*_(1,10)_ = 1.938; root diameter: *F*_(1,10)_ = 2.170; root volume: *F*_(1,10)_ = 12.570; all *p* < 0.001]. For *A. adenophora*, the root growth characteristics were significantly increased by inoculation treatment both in monoculture and mixture treatment, among which the root growth characteristics of BC + SC treatment were significantly higher (*p* < 0.05). Additionally, inoculation treatment of *R. amethystoides* in monoculture and mixture treatments with BC exhibited a significant increase in the root growth characteristics (*p* < 0.05), while inoculation treatments with SC and BC + SC resulted in a significant decrease in the root growth characteristics of *R. amethystoides* (*p* < 0.05).

### 3.4. Effects of competition and inoculum on *Ageratina adenophora* and *Rabdosia amethystoides* nutrient concentrations

**Figure 4:**
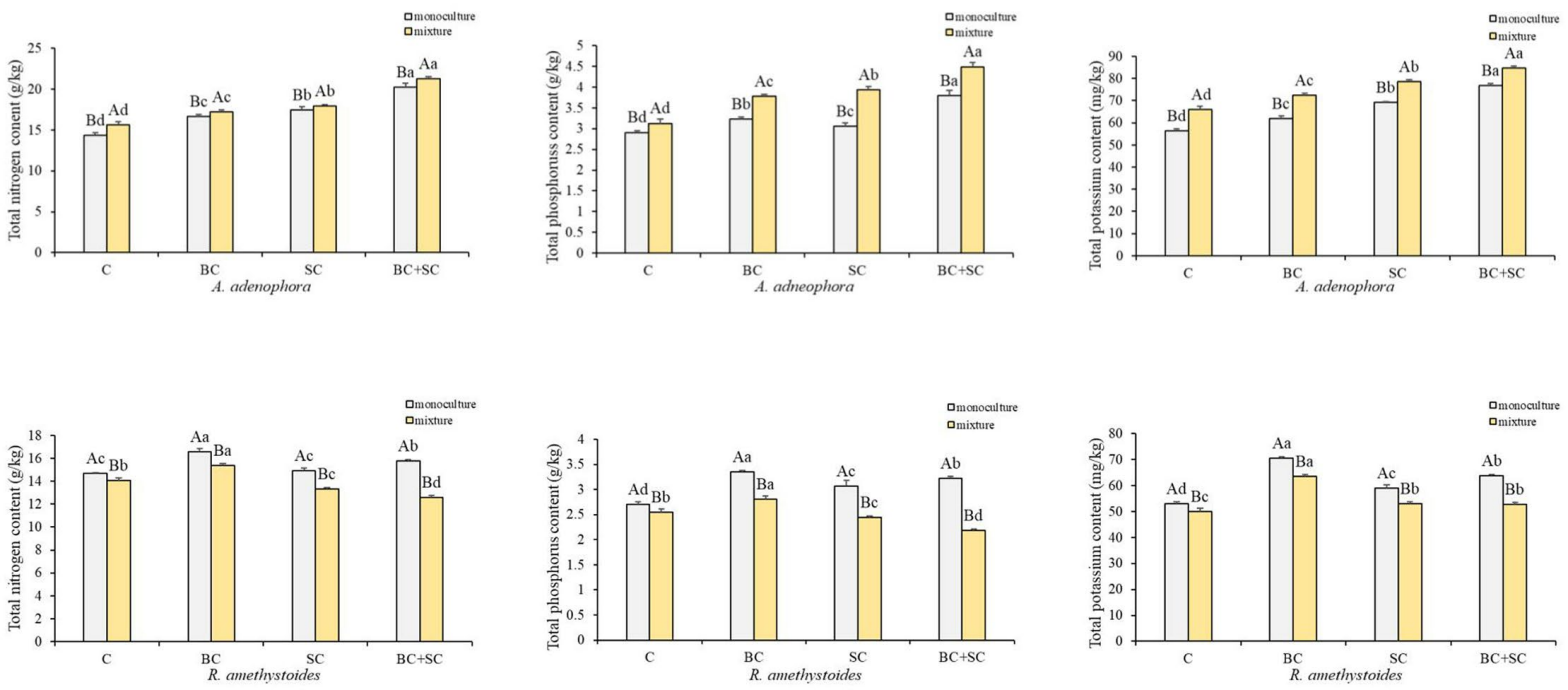
Effect of competition and inoculum on the nutrient concentrations of *A. adenophora* and *R. amethystoides*. C, control; BC, inoculated with *B. cereus*; SC, inoculated with *S. constrictum*; BC + SC, inoculated with *B. cereus* and *S. constrictum*. Different lowercase letters in lower case indicate significant differences between the four treatments at *p* < 0.05. Different uppercase letters indicate significant differences between the monoculture or mixture at *p* < 0.05. Error bars represent ± SE of mean (*n* = 6).

Competition effects on total N, P and K concentrations of *A. adenophora* and *R. amethystoides* were consistent with the trend in biomass (Figure 4 and Supplementary Table S1). *A. adenophora* in monoculture treatment showed significantly higher nitrogen, phosphorus and potassium concentrations than in mixture treatment [C: N: *F*_(1,10)_ = 1.285; P: *F*_(1,10)_ = 11.400; K: *F*_(1,10)_ = 1.556; all *p* < 0.001; BC: N: *F*_(1,10)_ = 0.010, *p* = 0.005; P: *F*_(1,10)_ = 1.560, *p* < 0.001; K: *F*_(1,10)_ = 0.890, *p* < 0.001; SC: N: *F*_(1,10)_ = 0.109, *p* = 0.032; P: *F*_(1,10)_ = 2.822, *p* < 0.001; K: *F*_(1,10)_ = 0.632, *p* < 0.001; BC + SC: N: *F*_(1,10)_ = 0.509, *p* = 0.001; P: *F*_(1,10)_ = 2.822, *p* < 0.001; K: *F*_(1,10)_ = 0.068, *p* < 0.001], while *R. amethystoides* in monoculture treatment had significantly increased nutrient concentrations than those in mixture treatment [C: N: *F*_(1,10)_ = 3.491, *p* < 0.001; P: *F*_(1,10)_ = 0.872, *p* = 0.001; K: *F*_(1,10)_ = 1.101; BC: N: *F*_(1,10)_ = 1.649; P: *F*_(1,10)_ = 4.085; K: *F*_(1,10)_ = 0.039; SC: N: *F*_(1,10)_ = 1.574; P: *F*_(1,10)_ = 3.467; K: *F*_(1,10)_ = 1.337; BC + SC: N: *F*_(1,10)_ = 0.434; P: *F*_(1,10)_ = 2.778; K: *F*_(1,10)_ = 0.448; all *p* < 0.001]. For *A. adenophora*, inoculation treatment significantly increased N, P, and K concentrations in monoculture and mixture treatments (*p* < 0.001). The nutrient contents of BC + SC treatment were significantly increased compared to other treatments (*p* < 0.001), and the N, P and K concentrations were increased by 41.47, 30.56, and 34.09% in monoculture treatment and 35.97, 43.26, and 28.61% in mixture treatment, respectively. For *R. amethystoides*, inoculation with BC treatment significantly enhanced N, P, and K concentrations in both monoculture and mixture treatments (*p* < 0.001), while inoculation of SC and BC + SC significantly increased the nutrient concentrations in monoculture treatment (*p* < 0.001) but decreased N and P concentrations in mixture treatment (*p* < 0.001). Inoculation with SC treatment reduced the N and P concentrations by 5.42 and 4.47%, and inoculation with BC + SC treatment reduced the N and P by 10.78 and 14.41% in mixture treatment, respectively.

### 3.5. Effects of competition and inoculum on AMF colonization and *Bacillus* density of *Ageratina adenophora* and *Rabdosia amethystoides*

**Figure 5:**
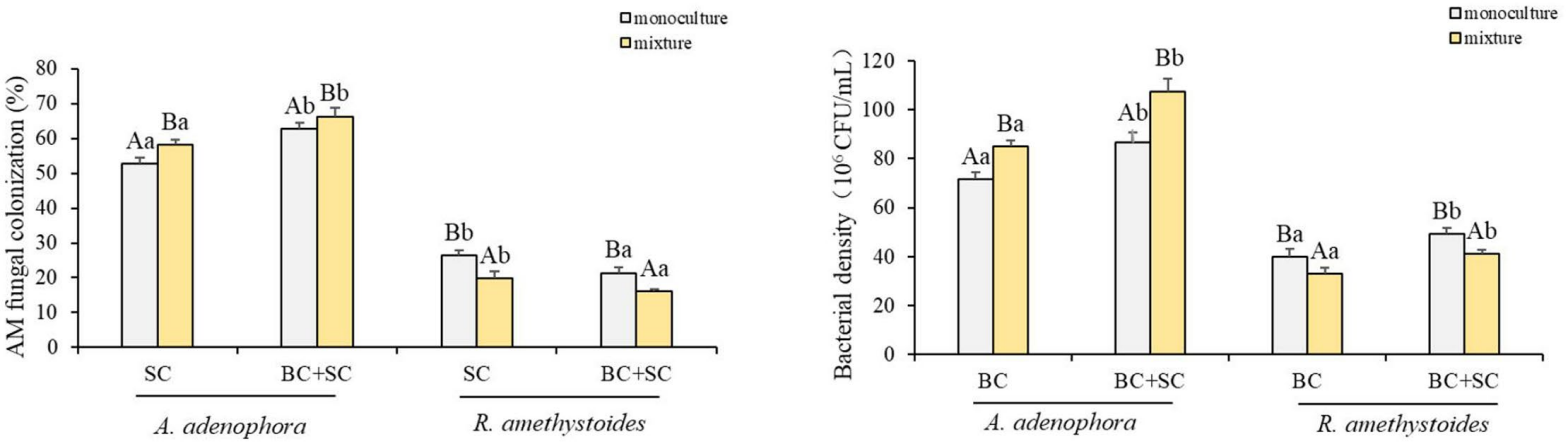
Effect of competition and inoculum on the AMF colonization and Bacterial density of *A. adenophora* and *R. amethystoides*. C, control; BC, inoculated with *B. cereus*; SC, inoculated with *S. constrictum*; BC + SC, inoculated with *B. cereus* and *S. constrictum*. Different lowercase letters in lower case indicate significant differences between the four treatments at *p* < 0.05. Different uppercase letters indicate significant differences between the monoculture or mixture at *p* < 0.05. Error bars represent ± SE of mean (*n* = 6).

*Ageratina adenophora* had a higher colonization rate than *R. amethystoides* in treatments with SC and BC + SC (Figure 5). Competition differentially affected the AMF colonization of the two plants. *A. adenophora* in mixture treatment showed a remarkably higher AMF colonization rate than that in monoculture treatment [SC: *F*_(1,10)_ = 0.003; BC + SC: *F*_(1,10)_ = 1.104; both *p* < 0.001], while *R. amethystoides* in mixture treatment had a significantly lower AMF colonization rate than that in monoculture treatment [SC: *F*_(1,10)_ = 0.064; BC + SC: *F*_(1,10)_ = 2.844; both *p* < 0.001]. *A. adenophora* inoculated with BC + SC had a significantly higher AMF colonization rate than that inoculated with SC in both monoculture and mixture treatments [monoculture: *F*_(3,20)_ = 102.150; mixture: *F*_(3,20)_ = 48.412, both *p* < 0.001]. However, *R. amethystoides* inoculated with SC had a significantly higher AMF colonization rate than that inoculated with SC in both monoculture and mixture treatment [monoculture: *F*_(3,20)_ = 32.194; mixture: *F*_(3,20)_ = 18.654; both *p* < 0.001].

*Bacillus* density in *A. adenophora* rhizosphere soil was significantly higher than in *R. amethystoides* rhizosphere soil in all treatments (Figure 5). In addition, the *Bacillus* density was significantly higher in *A. adenophora* rhizosphere soil in mixture treatment than that in monoculture treatment [BC: *F*_(1,10)_ = 0.363; BC + SC: *F*_(1,10)_ = 0.630; both *p* < 0.001]. However, *Bacillus* density was significantly higher in *R. amethystoides* rhizosphere soil in monoculture treatment than that in mixture treatment [BC: *F*_(1,10)_ = 1.875; BC + SC: *F*_(1,10)_ = 0.870; both *p* < 0.001]. Further, the *Bacillus* density in rhizosphere soil of *A. adenophora* and *R. amethystoides* in BC + SC treatment was significantly increased relative to that with BC treatment [*A. adenophora*: monoculture: *F*_(3,20)_ = 58.615; mixture: *F*_(3,20)_ = 90.504; *R. amethystoides*: monoculture: *F*_(3,20)_ = 34.690; mixture: *F*_(3,20)_ = 38.400; all *p* < 0.001].

### 3.6. Impact of competition and inoculum on soil characteristic

For *A. adenophora* monoculture treatment and *A. adenophora* and *R. amethystoides* mixture treatment, inoculation significantly increased nitrate-nitrogen (NO_3_^−^-N) and AP content in rhizosphere soil [Am: NO_3_^−^-N: *F*_(3,20)_ = 353.767; AP: *F*_(3,20)_ = 158.065; A + R: NO_3_^−^-N: *F*_(3,20)_ = 211.638; AP: *F*_(3,20)_ = 294.625; all *p* < 0.001, Table 1, and Supplementary Table S1]. Among them, NO_3_^−^-N and AP contents in the rhizosphere soil of BC inoculated treatment were significantly increased (*p* < 0.001). For *R. amethystoides* monoculture treatment, NO_3_^−^-N, AP, ammonium N (NH_4_^+^-N) and AK contents were significantly increased by the inoculation treatment [NO_3_^−^-N: *F*_(3,20)_ = 179.125; AP: *F*_(3,20)_ = 28.439; NH_4_^+^-N: *F*_(3,20)_ = 213.247; AK: *F*_(3,20)_ = 47.405; all *p* < 0.001], among which the available nutrient content of the rhizosphere soil of BC inoculation treatment was significantly increased (*p* < 0.001).

**Table 1:** Soil characteristics under different treatments.

Treatments		NO_3_^−^-N (μg/g)	NH_4_^+^-N (μg/g)	Available P (μg/g)	Available K (mg/g)
Am	C	13.190 ± 0.109 Bd	3.540 ± 0.103Ba	4.365 ± 0.086Ad	29.093 ± 1.327ABa
	BC	15.289 ± 0.714Aa	3.620 ± 0.106Ba	5.838 ± 0.190Aa	28.595 ± 1.618Ba
	SC	13.837 ± 0.179Ac	3.550 ± 0.070Ba	4.881 ± 0.090Ac	28.347 ± 1.543Aa
	BC + SC	14.343 ± 0.066Bb	3.515 ± 0.040Ba	5.155 ± 0.076Ab	28.117 ± 0.897Ba
A + R	C	13.652 ± 0.162Ac	3.732 ± 0.077Aa	4.337 ± 0.071Ad	29.713 ± 0.951Aa
	BC	15.518 ± 0.202Aa	3.708 ± 0.069Ba	6.012 ± 0.083Aa	30.015 ± 0.662ABa
	SC	13.847 ± 0.110Ac	3.542 ± 0.149Ba	4.923 ± 0.129Ac	28.645 ± 1.435Aa
	BC + SC	14.930 ± 0.102Ab	3.558 ± 0.152Ba	5.253 ± 0.106Ab	29.167 ± 1.285ABa
Rm	C	12.630 ± 0.092Cd	3.620 ± 0.164ABc	4.313 ± 0.085Ad	28.103 ± 0.571Bc
	BC	14.682 ± 0.269Ba	4.208 ± 0.078Aa	5.143 ± 0.058Ba	31.338 ± 0.269Aa
	SC	13.037 ± 0.120Bc	4.000 ± 0.078Ab	4.559 ± 0.049Bc	29.568 ± 0.459Ab
	BC + SC	14.302 ± 0.185Bb	3.990 ± 0.107Ab	4.933 ± 0.050Bb	30.330 ± 0.576Ab

C, control; BC, inoculated with *B. cereus*; SC, inoculated with *S. constrictum*; BC + SC, inoculated with *B. cereus* and *S. constrictum*; Am, *A. adenophora* monoculture; A + R: *A. adenophora* and R, amethystoides mixture; Rm, R. amethystoides monoculture. Different letters in lower case indicate significant differences between the four treatments at *p* < 0.05. Different uppercase letters indicate significant differences between the monoculture or mixture at *p* < 0.05.

### 3.7. Correlation of AMF colonization rate and the density of *Bacillus* with plant growth indicator and soil characteristics

**Figure 6:**
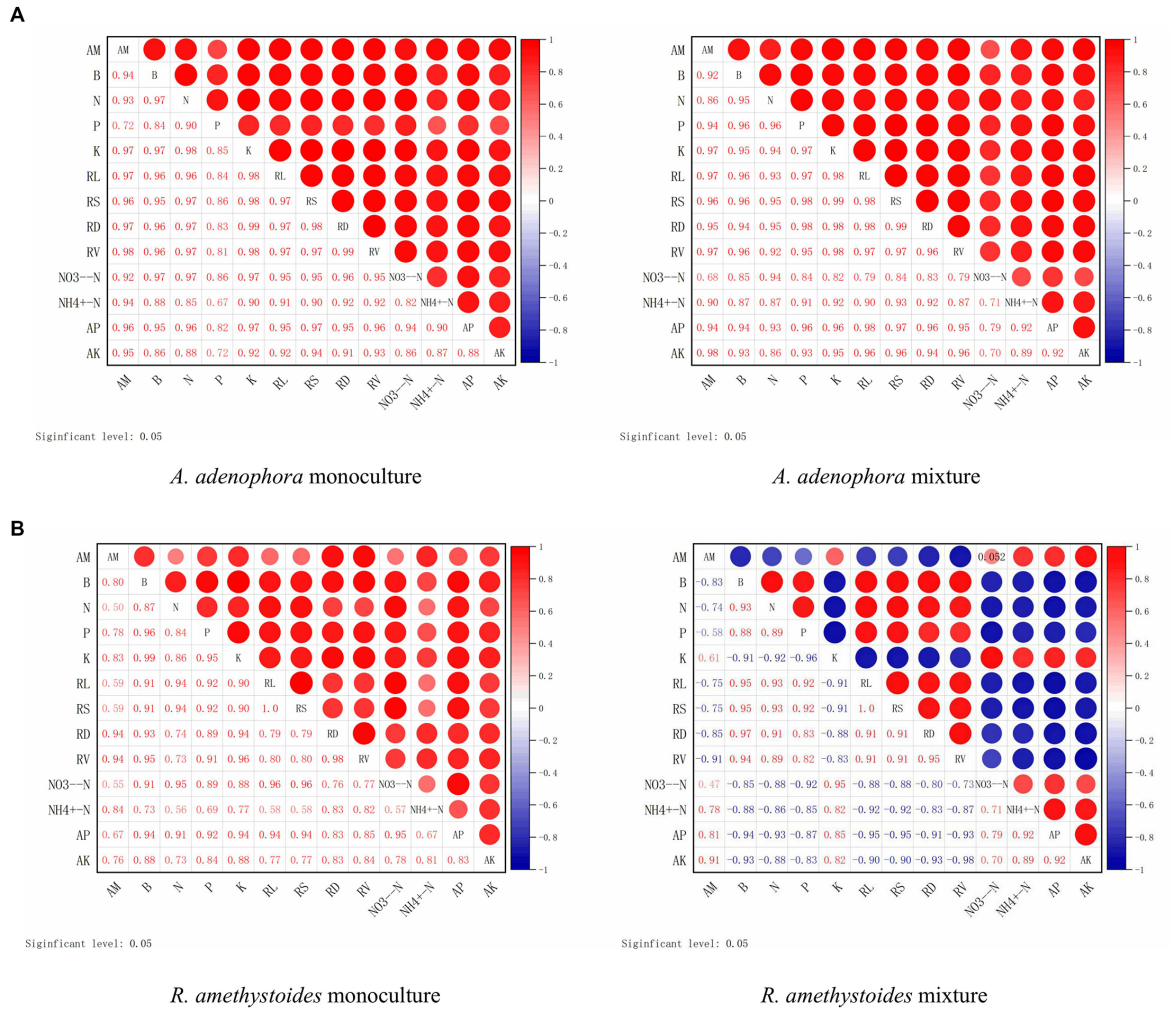
Correlation between root colonization by an arbuscular mycorrhizal fungus, *S. constrictum*, and plant growth indicators of *A. adenophora* **(A)** and *R. amethystoides* **(B)**. AM, AM colonization; B, biomass; RL, root length; RS, root surface area; RD, root diameter; RV, root volume. Red circles represent a positive correlation between root colonization and plant growth indicators. Larger circles represent a stronger correlation.

Here, we investigated the impact of monoculture and mixed cropping of *A. adenophora* with *R. amethystoides* on the colonization rate of *S. constrictum* and *B. cereus*, as well as their correlation with various growth parameters and nutrient concentrations in two different soil types, SC and BC + SC. Our findings indicated that *S. constrictum* colonization rate was positively correlated with biomass, total N, P and K concentrations, root growth characteristics, NO_3_^−^-N, and AP, but a negatively correlated with NH_4_^+^-N contents (except in SC inoculated monoculture treatment) and AK contents (Figure 6). In the mixture treatment of *A. adenophora* with *R. amethystoides* in SC and BC + SC treatment, *S. constrictum* colonization rate in *R. amethystoides* was negatively associated with biomass, total N and P concentrations and root growth characteristics, but positively correlated with total K concentration and soil characteristics (Figure 6).

**Figure 7:**
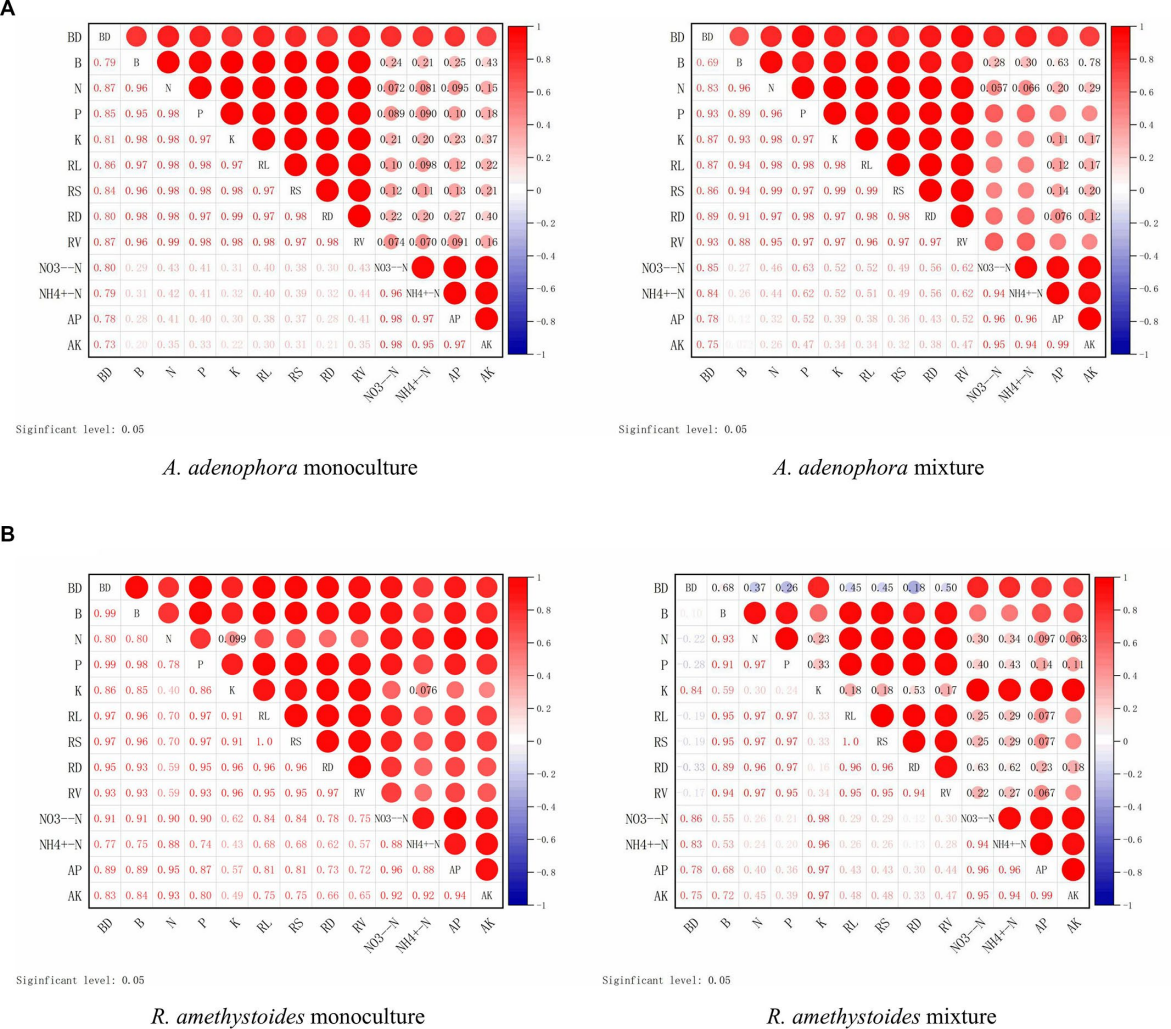
Correlation between the density of *B. cereus* (BC) and plant growth indicators of *A. adenophora* **(A)** and *R. amethystoides* **(B)**. AM, AM colonization; B, biomass; RL, root length; RS, root surface area; RD, root diameter; RV; root volume. Blue circles represent a negative correlation and red circles represent a positive correlation between root colonization and plant growth indicators. Larger circles represent a stronger correlation.

In both the monoculture and mixture treatments of *A. adenophora* with *R. amethystoides* in BC and BC + SC treatment, the density of *B. cereus* showed a significantly positive correlation with biomass, total N, P and K concentrations, root growth characteristics, NO_3_^−^-N and AP contents, but a negative correlation with NH_4_^+^-N and AK contents (Figure 7). In the mixture treatment with *A. adenophora* in BC + SC treatment, the density of *B. cereus* showed a significantly negative correlation with biomass, total N and P concentration and root growth characteristics but a positive correlation with total K concentration and soil characteristics (Figure 7).

## 4. Discussion

When invasive plants establish themselves in a new habitat, they tend to accumulate beneficial microorganisms (mainly comprising AMF and *Bacillus*) in their rhizosphere to promote their growth and facilitate their invasion in response to abiotic factors (i.e., nutrient deficiencies) and biotic factors (i.e., feeding by generalist insects) (Meisner et al., 2013; Fu et al., 2017; Mohanty et al., 2018). In this present study, we investigated the common AMF and *Bacillus* present in the rhizosphere soil of both the invasive plant (*A. adenophora*) and native plant (*R. amethystoides*) and the explored the effect of single microbes versus co-culture on the competitive growth of the two plants. We found that *S. constrictum* and *B. cereus* exerted different effects on the two plants. Compared with the control treatment, inoculation with *S. constrictum* enhanced *A. adenophora*’s competitive growth but inhibited that of *R. amethystoides*, while inoculation with *B. cereus* inhibited *A. adenophora* growth but facilitated that of *R. amethystoides* growth (Figure 2). *S. constrictum* and *B. cereus* significantly increased the AM fungal colonization rate in the root and *Bacillus* density in the rhizosphere of *A. adenophora* (Figure 5) and further improved its competitiveness (Figure 2). These results supported our hypothesis that *S. constrictum* and *B. cereus* can form a synergistic effect that further promotes *A. adenophora* competitiveness and invasion.

AMF is critical for the successful invasion of some exotic species (Bunn et al., 2015; Reinhart et al., 2017). AMF affects the competition of invasive species with native species by changing nutrient uptake (Zhang et al., 2017, 2018). In this study, the competitive ability of inoculated *S. constrictum* to *A. adenophora* was significantly increased, while that of *R. amethystoides* was significantly decreased compared with the control treatment, indicating that *S. constrictum* increased *A. adenophora* competition to *R. amethystoides* (Figure 2). Relative to the monoculture treatment, the N, P and K concentrations of *A. adenophora* were significantly increased, and those of *R. amethystoides* were concomitantly decreased when the two plant species were in interspecific competition (Figure 4). Our results were concordant with Shen et al. (2020), who found that AMF improved the competitive ability of *A. adenophora* against the native *A. annua* by significantly enhancing the N and P of the former. Therefore, AMFcan enhance *A. adenophora*’s competitiveness by providing more nutrients, which may be attributed to their different AMF colonization rates (Waller et al., 2016; Xia et al., 2021). Compared to monoculture treatment, the AMF colonization rate of *R. amethystoides* was significantly decreased, while the AMF colonization rate of *A. adenophora* was significantly increased when the two plant species were in interspecific competition. Chen et al. (2020) also found that competition reduced the AM colonization of native plants *Sesbania cannabina* and *Eupatorium chinense* by more than half when grown together with the invasive plants *Bidens pilosa* and *Eupatorium catarium*. AMF affects the competitiveness of both invasive and native species by altering their capacities for soil nutrient acquisition (Vogelsang and Bever, 2009; Pinzone et al., 2018). The correlation analysis results showed that in the mixture treatment, the increase in biomass, plant N, P, and K concentration and root growth characteristics of *A. adenophora*, as well as the decrease in biomass, plant N and P concentration and root growth characteristics of *R. amethystoides*, were significantly associated with AMF colonization rate when grown in the mixture treatment (Figure 6). These indicate that the colonization of roots by AMF can promote plant growth (Kong et al., 2022). Taken together, AMF contributes to *A. adenophora* out-competing native *R. amethystoides* through *S. constrictum*, which provides more nutrients to *A. adenophora* than to *R. amethystoides* with higher colonization in *A. adenophora*.

*Bacillus* is one of the rhizosphere-promoting bacteria for plants (Gupta et al., 2015; Gouda et al., 2018). It can directly release plant hormones, siderophores and ammonia, which provide soil nutrients through fixing N, and solubilizing P and K, thereby promoting plant growth (Ding et al., 2015; Khan et al., 2019). Moreover, it can also indirectly produce antimicrobial compounds to alleviate the inhibition of diverse pathogens in the plant (Alina et al., 2015; Khan et al., 2019). Our results also revealed that inoculation with *B. cereus* could significantly increase the nitrate nitrogen and AP contents in *A. adenophora* and *R. amethystoides* rhizosphere soil for plant growth (Table 1). Sun et al. (2021) showed that as the density of *B. cereus* increased, the soil available nutrient contents also increased. Roots directly affect the growth of aboveground parts and overall plant growth and development, with root growth and structure playing a key role in water and nutrient absorption (Comas et al., 2013; Ma et al., 2018). Here, *B. cereus* inoculation enhanced the root growth and nutrient absorption capacity of *A. adenophora* (Figure 3), thus increasing the N, P and K concentrations in the plants (Figure 4). A similar trend was also found in *R. amethystoides*. N and P have a positive synergistic effect, thus increasing the photosynthetic rate and improving the growth of plants (Schleuss et al., 2020). Our study also revealed that inoculation with *B. cereus* weakened the positive effect of interspecific competition on *A. adenophora* and alleviated the negative effect on *R. amethystoides* growth but did not change the competitive relationship between *A. adenophora* and *R. amethhystoides* (Figure 2). We also found that *B. cereus* inoculation led to significantly greater promotion on *R. amethystoides* biomass than *A. adenophora*, which may be due to the different effect of root exudates from the two plants on the growth-promoting effect of *Bacillus* (Sun et al., 2021, 2022). To understand the potential mechanisms, future studies may conduct a comparative analysis of the secondary metabolites of *A. adenophora* and *R. amethystoides* and their effect on *Bacillus*’s ability to solubilize phosphorus and fix nitrogen.

The combination of AMF and *Bacillus* is not only helpful to plant growth and resistance to various stresses but also benefits each other (Sangwan and Prasanna, 2021). In this present study, co-inoculation with *B. cereus* and *S. constrictum* treatments significantly increased the density of *B. cereus* in *A. adenophora* and *R. amethystoides* rhizosphere soil when the two species were grown in monoculture or together. Many *Bacillus* can be used as mycorrhizal helper bacteria (MHB), which is positively associated with root colonization and hyphal development of AMF. AMF can provide a habitat for bacteria and secrete mycelial secretions to promote the growth and development of PGPR (Zhang et al., 2018; Wang et al., 2022). Zhang et al. (2018) demonstrated that the hyphal exudates of AMF not only served as a carbon source for bacterial growth but also played a signaling role in triggering the bacteria-mediated organic phosphate mineralization process, which stimulated the expression of phosphatase genes in bacteria and released phosphatase. We revealed that inoculation with *B. cereus* isolated from the soil of mixture treatment of *A. adenophora* and *R. amethystoides* could increase soil nutrients in monoculture or mixture treatment, thereby increasing the N and P contents of both plants (Table 1). Several studies have shown that a suitable combination of inoculants with AMF and PGPR significantly affects plant growth compared with inoculations of the two alone (Zhang et al., 2014; Krishnamoorthy et al., 2016; Hidri et al., 2019). We found that the biomass, root growth characteristics, and nutrient contents of two plants with *B. cereus* and *S. constrictum* inoculation were significantly higher than those with single inoculation in *A. adenophora* or *R. amethystoides* monoculture. AMF mycelium recruits many bacteria, which provide functions that are absent from the AMF, thus further promoting the plant growth (Zhou et al., 2020; Jiang et al., 2021). In comparison with the monoculture, the AMF colonization rate of *A. adenophora* in the mixture of co-inoculation with *B. cereus* and *S. constrictum* treatments was significantly increased, while the AMF colonization rate of *R. amethystoides* was significantly reduced, which are consistent with the results of changes in nutrient content and growth in their respective plants. *B. cereus* inoculation increased the AMF colonization rate in *A. adenophora* rhizosphere soil in the SC + BC treatment and transported more activated nutrients for the plants. Meanwhile, the AMF colonization rate of *R. amethystoides* decreased when *A. adenophora* competed with *R. amethystoides*, which weakened the transport of available nutrients and inhibited the growth of *R. amethystoides*. Du et al. (2020) reported that AMF and *Bacillus* co-inoculation provided more N for *F. bidentis*, which enhanced the competitive advantage of *F. bidentis* over native *E. prostrata*. These results indicated that different functional microbial communities are involved in the underlying invasion mechanism, in which AMF may play a major role in the interspecific competition between invasive and native plants, and that *B. cereus*, as a mycorrhizal helper bacterial, promotes hyphal development and colonization of plant roots, thus helping the alien plant to compete over the native plant. Due to the few microbial species used in this study, the generality of our conclusion should be further tested by including more species. Moreover, it is necessary to continue to test the effects of AMF and *Bacillus* on the invasion ability of *A. adenophora* with various environmental factors in the future, such as non-sterile soil and different nutrient concentrations.

## 5. Conclusion

Our finding revealed that the symbiotic association of AMF and *Bacillus* with high content in the inter-rhizosphere soil of *A. adenophora* and *R. amethystoides* increased the competitive advantage of *A. adenophora*. However, *S. constrictum* and *B. cereus* played different roles in the invasion of *A. adenophora*. *S. constrictum* provided competitive advantages with different AM fungal colonization rates in the roots of *A. adenophora* and *R. amethystoides*, which resulted in more nutrient supply to invasive plants, while *B. cereus* may have activated more soil nutrients and promoted the hyphal development and colonization of plant roots. Overall, these findings enhanced our understanding on the role of AMF and bacteria in invasion by *A. adenophora*.

## Data availability statement

The original contributions presented in the study are included in the article/Supplementary material, further inquiries can be directed to the corresponding author.

## Author contributions

FG, ED, and YC designed the research. YL and RH collected the samples. ED and YHL performed the experiments. ZS and YHL performed bioinformatic and statistical analyses. ED and YC wrote the first draft. ZS and FG reviewed the manuscript. All authors contributed to the article and approved the submitted version.

## Funding

This work was supported by the Yunnan Eco-Friendly Food International Cooperation Research Center (YEFICRC) Project of Yunnan Provincial of Yunnan Provincial Key Program (Grant No. 2019ZG00910), National Key Research and Development Program of China (2021YFD1400200), and Scientific Research Foundation of Education Department of Yunnan Province (2022Y224).

## Conflict of interest

The authors declare that the research was conducted in the absence of any commercial or financial relationships that could be construed as a potential conflict of interest.

## Publisher’s note

All claims expressed in this article are solely those of the authors and do not necessarily represent those of their affiliated organizations, or those of the publisher, the editors and the reviewers. Any product that may be evaluated in this article, or claim that may be made by its manufacturer, is not guaranteed or endorsed by the publisher.
