# Supplementary material for: Effects of Septoglomus constrictum and Bacillus cereus on the competitive growth of Ageratina adenophora
Source: Front Microbiol. 2023 Jun 2;14:1131797. doi: 10.3389/fmicb.2023.1131797 (PMC10272390; doi:10.3389/fmicb.2023.1131797)
Supplement: Supplementary file 1 [file Table_1.DOCX]

Supplementary Material

## 1. Supplementary Table

Table S1 Two-way ANOVAs of the effects of inoculation with inoculation treatment and plant competition on the growth indicators of *A. adenophora*

| Plant | Parameters | Inoculation treatment | | Plant competition treatment | | Inoculum*competition treatment | |
| --- | --- | --- | --- | --- | --- | --- | --- |
|  |  | *F* | *P* | *F* | *P* | *F* | *P* |
| *A. adenophora* | Biomass | 190.153 | <0.001*** | 200.381 | <0.001*** | 40.261 | <0.001*** |
|  | N | 611.232 | <0.001*** | 71.641 | <0.001*** | 3.885 | <0.001*** |
|  | P | 409.452 | <0.001*** | 658.145 | <0.001*** | 37.331 | <0.001*** |
|  | K | 1033.894 | <0.001*** | 1281.722 | <0.001*** | 3.981 | 0.014* |
|  | Root length | 508.676 | <0.001*** | 212.169 | <0.001*** | 0.231 | 0.874 |
|  | Root surface area | 649.606 | <0.001*** | 140.759 | <0.001*** | 2.215 | 0.101 |
|  | Root diameters | 468.926 | <0.001*** | 129.254 | <0.001*** | 2.981 | 0.043* |
|  | Root volume | 328.591 | <0.001*** | 89.984 | <0.001*** | 1.096 | 0.362 |
|  | NO_3_^-^-N | 518.285 | <0.001*** | 69.793 | <0.001*** | 10.953 | <0.001*** |
|  | NH_4_^+^-N | 4.647 | 0.007** | 7.046 | 0.011* | 2.053 | 0.122 |
|  | Available P | 426.370 | <0.001*** | 5.050 | 0.030* | 1.809 | 0.161 |
|  | Available K | 1.604 | 0.204 | 5.460 | 0.025 | 0.458 | 0.713 |
| *R. amethystoides* | Biomass | 518.806 | <0.001*** | 3405.101 | <0.001*** | 403.545 | <0.001*** |
|  | N | 264.167 | <0.001*** | 916.393 | <0.001*** | 106.159 | <0.001*** |
|  | P | 87.966 | <0.001*** | 1181.665 | <0.001*** | 158.121 | <0.001*** |
|  | K | 493.606 | <0.001*** | 431.029 | <0.001*** | 233.070 | <0.001*** |
|  | Root length | 136.260 | <0.001*** | 964.999 | <0.001*** | 210.875 | <0.001*** |
|  | Root surface area | 136.260 | <0.001*** | 964.999 | <0.001*** | 210.875 | <0.001*** |
|  | Root diameters | 71.751 | <0.001*** | 1575.475 | <0.001*** | 271.815 | <0.001*** |
|  | Root volume | 116.684 | <0.001*** | 2057.722 | <0.001*** | 297.679 | <0.001*** |
|  | NO_3_^-^-N | 381.960 | <0.001*** | 297.428 | <0.001*** | 2.836 | 0.05 |
|  | NH_4_^+^-N | 75.959 | <0.001*** | 0.498 | 0.484 | 1.810 | 0.161 |
|  | Available P | 490.178 | <0.001*** | 269.308 | <0.001*** | 53.260 | <0.001*** |
|  | Available K | 238.512 | <0.001*** | 35.556 | <0.001*** | 13.412 | <0.001*** |

*: *P*<0.05, **: *P*<0.01, ***: *P*<0.001
